# Medicare Advantage Financing and Quality in Puerto Rico vs the 50 US States and Washington, DC

**DOI:** 10.1001/jamahealthforum.2022.3073

**Published:** 2022-09-16

**Authors:** Thomas Roberts, Zirui Song

**Affiliations:** 1 Department of Medicine, Massachusetts General Hospital, Boston; 2 Department of Medical Oncology, Dana-Farber Cancer Institute, Boston, Massachusetts; 3 Department of Health Care Policy, Harvard Medical School, Boston, Massachusetts; 4 Center for Primary Care, Harvard Medical School, Boston, Massachusetts

## Abstract

**Importance:**

More than 70% of Medicare beneficiaries in Puerto Rico are enrolled in a Medicare Advantage (MA) plan. Evidence of MA plan payments and quality in Puerto Rico compared with the 50 US states and Washington, DC (hereafter referred to as US mainland), is lacking, notably after implementation of the Patient Protection and Affordable Care Act (ACA).

**Objective:**

To compare MA plan payments and quality in Puerto Rico with those in the US mainland and to evaluate how differences between MA plans in Puerto Rico and the US mainland changed after ACA implementation.

**Design, Setting, and Participants:**

This cohort study used publicly available data on MA plans from January 1, 2006, to December 31, 2019, from the Centers for Medicare & Medicaid Services. Data analysis was performed from October 2019 to February 2022.

**Exposures:**

Medicare Advantage plans in Puerto Rico and implementation of the ACA.

**Main Outcomes and Measures:**

Primary outcomes were risk-standardized federal benchmark payments (the amount offered by the federal government for insuring a beneficiary of average risk), risk-standardized plan bids (a plan’s asking price for a beneficiary of average risk), and rebates received by plans. Additional outcomes included risk-adjusted benchmarks, risk-adjusted bids, actual plan payment, and aggregate plan quality ratings (star ratings). A difference-in-differences analysis examined differential changes in plan payments in Puerto Rico vs the US mainland after ACA implementation.

**Results:**

Before ACA implementation, 211 MA plans in Puerto Rico and 13 899 plans in the US mainland were included. After ACA implementation, 433 MA plans in Puerto Rico and 29 515 plans in the US mainland were included. Before ACA implementation, risk-standardized benchmarks were 33% lower for MA plans in Puerto Rico than plans in the US mainland ($556.73 [95% CI, $551.82-$561.64] vs $831.15 [95% CI, $828.55-$833.75] per beneficiary per month [PBPM]). This gap increased to 38% after ACA implementation ($540.58 [95% CI, $536.86-$544.32] vs $869.31 [95% CI, $868.21-$870.42] PBPM). Risk-standardized plan bids in Puerto Rico were 46% lower before ACA implementation and 43% lower after ACA implementation compared with those in the US mainland. Rebates in Puerto Rico decreased from $168.50 (95% CI, $163.57-$173.42) PBPM before ACA implementation to $93.39 (95% CI, $89.51-$97.27) PBPM after ACA implementation, a decrease of $75.11 PMPM compared with a decrease of $2.05 PMPM in the US mainland. Plans in Puerto Rico received increased quality bonus payments, and the mean (SD) risk score for plans in Puerto Rico increased to 1.55 (0.31) after ACA implementation, which increased risk-adjusted benchmarks and actual plan payments, offsetting the widening payment disparity.

**Conclusions and Relevance:**

This cohort study found that after implementation of the ACA, federal benchmark payment amounts decreased in Puerto Rico compared with the US mainland. Responses by MA plans in Puerto Rico, including increased quality bonus payments and risk scores, offset this payment reduction, although actual plan payments in Puerto Rico were lower than those in the US mainland.

## Key Points

**Question:**

How do Medicare Advantage (MA) plan payments and quality ratings differ between Puerto Rico and the 50 US states and Washington, DC, and how did they change after implementation of the Patient Protection and Affordable Care Act (ACA)?

**Findings:**

In this cohort study with a difference-in-differences analysis of MA plans in Puerto Rico and the 50 US states and Washington, DC, MA plans in Puerto Rico were offered lower federal benchmark payments. After ACA implementation, plan payments differentially decreased in Puerto Rico, but responses by MA plans partially offset these decreases.

**Meaning:**

In this study, MA plans in Puerto Rico received lower payments than those in the 50 US states and Washington, DC, and this gap widened after ACA implementation.

## Teaser

This cohort study analyzes differences in Medicare Advantage plan payments and quality ratings between Puerto Rico and the 50 US states and Washington, DC, and changes in these difference after Patient Protection and Affordable Care Act implementation.

## Introduction

Puerto Rico, a US territory since 1917, experiences unique economic and health challenges. The 3.2 million US citizens living in the territory have a median household income of $19 343, less than one-third of the $60 336 US median household income, and 46% of individuals in Puerto Rico live below the federal poverty level.^1,2^ The economy in Puerto Rico is shrinking, the territory is experiencing an ongoing debt crisis, and recent natural disasters damaged infrastructure and caused large numbers of deaths.^3,4,5,6^ More than 500 000 individuals in Puerto Rico, including many health care professionals, emigrated to the 50 US states and Washington, DC (hereafter referred to as US mainland) during the past decade.^7,8^ Health care infrastructure is limited in areas outside Puerto Rico’s capital, San Juan, and 72 of the territory’s 78 counties are considered medically underserved.^4,7^ Prior work has shown lower rates of cancer screening and vaccinations among individuals living in Puerto Rico and worse performance on many quality measures compared with those for Hispanic individuals in the US mainland.^7,9,10^

Puerto Rico has legal restrictions not present in the US mainland. Residents pay many federal taxes, including Medicare payroll taxes, but they do not receive important federal benefits, such as Supplemental Security Income and the Medicare Part D low-income subsidy.^3^ More than 65% of individuals living in Puerto Rico receive health care through Medicaid and Medicare, but federal funding for these programs in US territories has restrictions not present in the US mainland.^11,12,13^ For example, in Puerto Rico, Medicaid is funded by a federal block grant, set in 2018 at $357.8 million for 1.5 million enrollees.^2,14^ As a comparison, in 2018, Mississippi received $4.4 billion in federal Medicaid funding for 699 143 enrollees.^15^ As a result, the health care system in Puerto Rico has required more than $16 billion of ad hoc supplemental funds since 2010.^16,17^ Owing, in part, to these restrictions, the annual per capita health care expenditures in Puerto Rico were $3065 in 2015 compared with $9515 in the US mainland in the same year.^7,18^

Medicare Advantage (MA) is a privately administered, capitated Medicare program. Medicare Advantage plan payments are determined in a bidding process.^19^ First, the federal government publishes benchmark payment rates—the amount that Medicare determines is a reasonable target amount, based on historical spending, for an MA plan to insure an average-risk beneficiary—for each county. Second, MA plans submit bids to the federal government that represent the amount the plan determines it needs for insuring an average-risk beneficiary. If the bid is lower than the benchmark, the plan receives a rebate (a portion of the difference between the benchmark and the bid), which it must use to offer additional benefits or reduce enrollees’ Medicare Part B or Part D cost sharing. The MA bidding process is the same in Puerto Rico and in the US mainland.

Medicare Advantage covers more than 70% of Medicare beneficiaries in Puerto Rico and accounts for approximately 50% of health care spending in the territory.^20,21^ Medicare Advantage enrollment increased in Puerto Rico in the 2000s in response to the Puerto Rican government promoting MA as a way to reduce costs. In addition, legislation increased federal benchmarks in Puerto Rico, which attracted insurers to the territory.^13,22^ However, in 2009, most county benchmarks in Puerto Rico were set at floor rates, established by the Medicare Modernization Act of 2003, that were 180% of estimated Medicare fee-for-service (FFS) spending. Despite these increased rates, benchmarks in Puerto Rico remained substantially lower than benchmarks in the US mainland.^13,23^

The Patient Protection and Affordable Care Act (ACA) aimed to reduce MA spending to help finance insurance expansions in the ACA Marketplace and in Medicaid. This reduction was implemented through redefining federal benchmarks. In the highest-spending quartile of counties, benchmarks based on traditional Medicare spending were set at 95% of that spending, and benchmarks in the lowest-spending quartile of counties, which included Puerto Rico, were set at 115% of traditional Medicare spending. The ACA also introduced quality incentives into plan payments based on star ratings composed of process, access, outcomes, and patient-experience measures; a plan could receive increases in its payments that could help offset the benchmark reductions.

In Puerto Rico, the ACA was expected to decrease MA benchmarks, and these projected decreases raised concern about whether traditional Medicare spending accurately reflected spending for MA beneficiaries given the disproportionately large share of beneficiaries in Puerto Rico enrolled in MA. Additional concerns emerged about data quality issues, high rates of zero-dollar beneficiaries in Puerto Rico, and legal exceptions for US territories.^12,24^ After ACA implementation, the Centers for Medicare & Medicaid Services (CMS) made several rule changes to address these concerns, such as removing zero-dollar claimants from MA benchmark calculations, modifying risk-adjustment calculations, and adjusting the geographic practice cost index (GPCI) in Puerto Rico (Table 1),^25,26,27,28,29,30,31,32^ although these changes were projected to only moderately increase MA funding in Puerto Rico.^24,30^

**Table 1.** Notable Changes to Medicare Advantage Payment Policies Affecting Puerto Rico From 2013 to 2019

Year	Stated problem	Description of change^a^
2013	Medicare beneficiaries in Puerto Rico were not automatically enrolled in Part B, and residents of Puerto Rico could not access the Medicare Savings Programs to help pay for Part B.	CMS updated benchmark calculations in Puerto Rico to include only traditional Medicare beneficiaries with Parts A and B coverage.^25,26^
2016	Medicare inpatient hospital payments were lower than those in the 50 US states; Washington, DC; and other territories.	The Consolidation Appropriations Act, 2016, set inpatient payments in Puerto Rico at national averages, the standard in other US territories.^27^
2017	There was a higher rate of zero-dollar claimants among traditional Medicare enrollees in Puerto Rico.	CMS made adjustments to benchmark calculations to account for high rates of zero-dollar claimants in Puerto Rico.^28^
2017	Medicare beneficiaries in Puerto Rico were not eligible for the Part D LIS.	CMS implemented an adjustment factor for quality scores of plans in Puerto Rico to account for the absence of LIS in Puerto Rico; CMS also reduced the weights applied to quality measures that could be impacted by the absence of the LIS in Puerto Rico.^28^
2017	GPCI was lower in Puerto Rico compared with the mean in the 50 US states; Washington, DC; and other territories.	CMS increased GPCI components in Puerto Rico with the plan for parity with national averages, the standard in other US territories, beginning in 2018.^29^
2017	Puerto Rico Medicare Advantage plans had high rates of dually eligible enrollees, and risk-adjustment models did not capture the full costs of care for these beneficiaries.	CMS implemented a revised risk-adjustment model to account for high rates of dually eligible enrollees^30^; this change was implemented nationally and was projected to have a larger effect on risk scores of plans in Puerto Rico.
2018	Double bonus payments were not available in Puerto Rico.	Medicare expanded eligibility for double bonus payments to Puerto Rico.^31^
2019	Puerto Rico was affected by several natural disasters that may have affected plans’ performance on quality measures.	Medicare adjusted quality measures and star ratings for plans in Puerto Rico to account for natural disasters.^32^

Abbreviations: CMS, Centers for Medicare & Medicaid Services; GPCI, geographic practice cost index; LIS, low-income subsidy.

^a^ This list is not inclusive of all changes made during this period.

To date, little empirical evidence on MA payments in Puerto Rico exists despite its importance in the territory. Moreover, evidence is lacking on how payments in Puerto Rico changed after ACA implementation. This study describes MA plan characteristics, federal benchmarks, plan bids, and plan rebates in Puerto Rico compared with those in the US mainland from 2006 to 2019. The study also examined how MA plans in Puerto Rico changed after ACA implementation compared with plans in the US mainland.

## Methods

### Data Sources and Population

In this cohort study, we examined publicly available data on MA plans from the CMS from January 1, 2006, to December 31, 2019. We obtained MA enrollment from CMS monthly enrollment reports from July of each year studied,^33^ and MA penetration was obtained from CMS penetration reports.^34^ County benchmarks were obtained from CMS rate books.^35^ Plan bids, hierarchical condition category (HCC) risk scores, and rebates were obtained from MA payment data published by CMS.^36^ The star ratings of Medicare plans were obtained from annual Medicare star ratings spring releases.^37^ Medicare unit prices for selected physician services and GPCI values were obtained from the Medicare Physician Fee Schedule.^38^ This study was exempt from review by the Harvard Medical School institutional review board, and informed consent was waived because it used aggregate, publicly available data with no identifying information. This study followed the Strengthening the Reporting of Observational Studies in Epidemiology (STROBE) reporting guideline.^39^

We restricted analysis to the main types of MA plans: health maintenance organization, local preferred provider organization, and private FFS. We also included special needs plans (SNPs). We excluded regional preferred provider organization, employer group waiver, Program of All-Inclusive Care for the Elderly, pilot and demonstration plans, and plans in US territories other than Puerto Rico. We also excluded the small number of plan-county-year records in US mainland counties from plans operating in Puerto Rico because the records were determined to be administrative errors. As summary statistics, we reported total enrollment and penetration including enrollees from all MA plan types.

### Outcomes

The 3 primary outcomes were risk-standardized plan benchmarks (the amount offered by the federal government for insuring a beneficiary of average risk), risk-standardized plan bids (a plan’s asking price for a beneficiary of average risk), and plan rebates (amount the government gives to plans for enhancing coverage or reducing patient cost sharing). Risk-standardized thus refers to a beneficiary of average risk. We calculated risk-standardized plan benchmarks as the mean benchmark for an average-risk beneficiary across the counties where the plan was active, weighted by enrollees in each county. Risk-standardized plan bids and plan rebates were the values reported for each plan in MA payment data.

Additional outcomes included risk-adjusted plan benchmarks, risk-adjusted plan bids, actual plan payments, and MA star ratings. Risk-adjusted benchmarks and bids were calculated for each plan as the product of the risk-standardized value and the plan’s CMS HCC risk score. Actual plan payments were the sum of the risk-adjusted bid and the plan rebate. For plan star ratings, we used the values reported by CMS. The definitions of outcomes, including data sources used for each outcome, are detailed in eTable 1 in the Supplement.

### Statistical Analysis

In descriptive analyses, we compared plan characteristics in Puerto Rico with those in the US mainland using unpaired *t* tests. To assess whether changes in plan benchmarks, bids, rebates, and actual plan payments in Puerto Rico differentially changed from those in the US mainland after ACA implementation, we used a difference-in-differences approach of the following form:

*Y_i,t_* = β_0_ + β_1_*PR_i_* + β_2_*ACA_t_* + β_3_(*PR_i_* ∗ *ACA_t_*) + β_4_*Riskscore* + β_5_*Plantype* + *μ_i_*,

where *Y_i,t_* denotes the outcome of interest for plan *i* in year *t*, *PR_i_* indicates whether the plan is in Puerto Rico or the US mainland, and *ACA_t_* indicates before or after ACA implementation. The interaction between the Puerto Rico and ACA indicators produced the coefficient of interest β_3_. The models adjusted for plan risk score and plan type. In our base specification, we defined the periods before and after ACA implementation nonparametrically using year indicators, in which each year indicator interacted with the Puerto Rico indicator. We assessed differences in trends before ACA implementation and mean effects after ACA implementation without imposing a linear (or other) functional form across years.

We considered 2010 to be a washout period owing to ACA implementation, because changes in the formula for federal payments to MA plans were introduced that year. Thus, 2010 was excluded from the main analysis. We tested the robustness of results to including 2010 in the post–ACA implementation period and excluding 2010 and 2011. Additional sensitivity analyses included only MA plans with the lowest 25% of benchmarks in the US mainland and the addition of a variable indicating whether a plan was an SNP. Data analyses were conducted from October 2019 to February 2022 using Stata, version 15.1 (StataCorp LLC).

## Results

### MA Participation

Medicare Advantage penetration in Puerto Rico increased from 60.1% in 2008 to 72.4% in 2019, with high rates of enrollment in all counties (range, 60.9%-92.0%). Medicare Advantage penetration in the US mainland increased from 24.5% in 2008 to 34.9% in 2019 (eFigures 1 and 2 in the Supplement). This study included 211 MA plans in Puerto Rico and 13 899 plans in the US mainland before ACA implementation and 433 MA plans in Puerto Rico and 29 515 plans in the US mainland after ACA implementation. Characteristics of counties and plans before and after ACA implementation are summarized in Table 2. Most counties in Puerto Rico (77 of 78 [99%]) had local MA plans before ACA implementation, and 100% had MA plans after ACA implementation. In the US mainland, 1726 counties (55%) had at least 1 local MA plan before ACA implementation compared with 2327 counties (74%) after ACA implementation.

**Table 2.** Characteristics of the MA Program in Puerto Rico and the US Mainland Before and After ACA Implementation^a^

Characteristic	Before ACA implementation		After ACA implementation	
	Puerto Rico	US mainland	Puerto Rico	US mainland
MA penetration, %^b^	61.60	22.80	72.70	31.60
Counties with MA plans, No./total No. (%)	77/78 (99)	1726/3143 (55)	78/78 (100)	2327/3143 (74)
County-level measures, mean (95% CI)^c^				
MA enrollees	4093 (3603-4584)	2174 (2003-2345)	5831 (5401-6263)	3680 (3502-3859)
MA insurers	7.64 (7.44-7.85)	3.51 (3.47-3.57)	4.63 (4.57-4.69)	2.68 (2.65-2.70)
MA plans	21.29 (20.33-22.25)	8.05 (7.88-8.22)	25.49 (25.00-25.97)	8.11 (8.00-8.22)
Total MA plans, No.	211	13 899	433	29 519
Plan-level measures, mean (95% CI)				
Risk score^d^	1.25 (1.19-1.30)	1.02 (1.01-1.03)	1.42 (1.37-1.47)	1.08 (1.07-1.08)
Star rating^e^	NA	NA	3.44 (3.31-3.56)	3.89 (3.87-3.92)
Enrollees^f^	6052 (4400-7706)	1899 (1807-1990)	10 888 (9094-12 683)	4205 (4095-4316)

Abbreviations: ACA, Patient Protection and Affordable Care Act; CMS, Centers for Medicare & Medicaid Services; MA, Medicare Advantage.

^a^ US mainland refers to the 50 states and Washington, DC. The period before ACA implementation was from 2006 to 2009, and the period after ACA implementation was from 2011 to 2019.

^b^ Penetration of all MA plan types in all county and county equivalents. Includes data from 2008 through 2019.

^c^ Means are for counties with at least 1 MA plan of the types included in this analysis (health maintenance organization, preferred provider organization, and private fee for service).

^d^ The hierarchical condition category risk scores published by CMS for each plan.

^e^ Summary statistics based on data from 2012 through 2019, the years that star ratings were published by the CMS.

^f^ Means were not weighted by the number of enrollees in each plan.

Before ACA implementation, counties in Puerto Rico had a mean of 7.64 (95% CI, 7.44-7.85) insurers and 21.29 (95% CI, 20.33-22.25) plans per county, exceeding the mean 3.51 (95% CI, 3.47-3.57) insurers and 8.05 (95% CI, 7.88-8.22) plans among counties with MA plans in the US mainland. After ACA implementation, the mean number of insurers per county in Puerto Rico decreased to 4.63 (95% CI, 4.57-4.69) compared with 2.68 (95% CI, 2.65-2.70) insurers per county in the US mainland. The mean number of plans per county among counties with MA plans was 25.49 (95% CI, 25.00-25.97) in Puerto Rico and 8.11 (95% CI, 8.00-8.22) in the US mainland (eFigure 3 in the Supplement).

### Plan Payments

Before ACA implementation, the risk-standardized benchmarks for MA plans in Puerto Rico were 33% lower than those in the US mainland ($556.73 [95% CI, $551.82-$561.64] vs $831.15 [95% CI, $828.55-$833.75] per beneficiary per month [PBPM]) (Table 3). This difference increased to 38% after ACA implementation ($540.58 [95% CI, $536.86-$544.32] vs $869.31 [95% CI, $868.21-$870.42] PBPM). Traditional Medicare prices for 12 commonly used Current Procedural Terminology codes were approximately 16% lower in Puerto Rico compared with the national payment amount (eTable 2 in the Supplement), reflecting the lower GPCI in Puerto Rico before 2018 (eFigure 4 in the Supplement), the year when CMS set the GPCI fee components for Puerto Rico at national averages.

**Table 3.** Changes in Medicare Advantage Plan Bids, Benchmarks, and Rebates After Implementation of the ACA

Measure	Puerto Rico			US mainland^a^			Differential change, $	
	Before ACA implementation, $ (95% CI)	After ACA implementation, $ (95% CI)	Change, $	Before ACA implementation, $ (95% CI)	After ACA implementation, $ (95% CI)	Change, $	Unadjusted^b^	Adjusted (95% CI)^c^
Risk-standardized benchmark^d^	556.73 (551.82 to 561.64)	540.58 (536.86 to 544.32)	−16.15	831.15 (828.55 to 833.75)	869.31 (868.21 to 870.42)	38.16	−54.31	−69.85 (−82.04 to −57.66)^d^
Risk-standardized bid	380.01 (373.95 to 386.07)	418.70 (412.30 to 425.10)	38.69	700.80 (699.31 to 702.30)	732.42 (731.42 to 733.41)	31.62	7.07	−13.60 (−36.23 to 9.03)
Rebate	168.50 (163.57 to 173.42)	93.39 (89.51 to 97.27)	−75.11	92.07 (90.77 to 93.37)	90.02 (89.13 to 90.91)	−2.05	−73.06	−63.70 (−82.73 to −44.68)^d^
Risk-adjusted benchmark^e^	694.42 (677.85 to 710.99)	767.49 (752.29 to 782.70)	73.07	852.90 (848.16 to 857.64)	939.59 (936.13 to 943.04)	86.69	−13.62	−22.83 (−77.75 to 32.09)
Risk-adjusted bid^e^	476.24 (461.45 to 491.02)	607.22 (588.57 to 625.88)	130.98	714.60 (711.53 to 717.70)	790.67 (787.76 to 793.58)	76.07	54.91	51.00 (−2.82 to 104.82)
Actual plan payment^e^	644.73 (629.83 to 659.64)	700.61 (684.51 to 716.71)	55.88	806.60 (802.99 to 810.22)	880.69 (877.58 to 883.81)	74.09	−18.21	−12.01 (−65.66 to 41.64)

Abbreviation: ACA, Patient Protection and Affordable Care Act.

^a^ US mainland refers to the 50 states and Washington, DC.

^b^ Calculated as the change in the outcome after ACA implementation in the US mainland minus the change in the outcome in Puerto Rico after ACA implementation.

^c^ Differential changes after ACA implementation based on the difference-in-differences models, which were adjusted for plan type and plan risk score.

^d^ Trends in the risk-standardized benchmark between Puerto Rico and the US mainland were not parallel before ACA implementation (mean relative change, −$15.31; 95% CI, $−18.70 to $−11.93). Therefore, although the difference increased after ACA implementation, the entire differential change could not be attributed to the ACA.

^e^ Given that these outcomes were risk adjusted, the model did not further adjust for risk.

**Figure 1.**
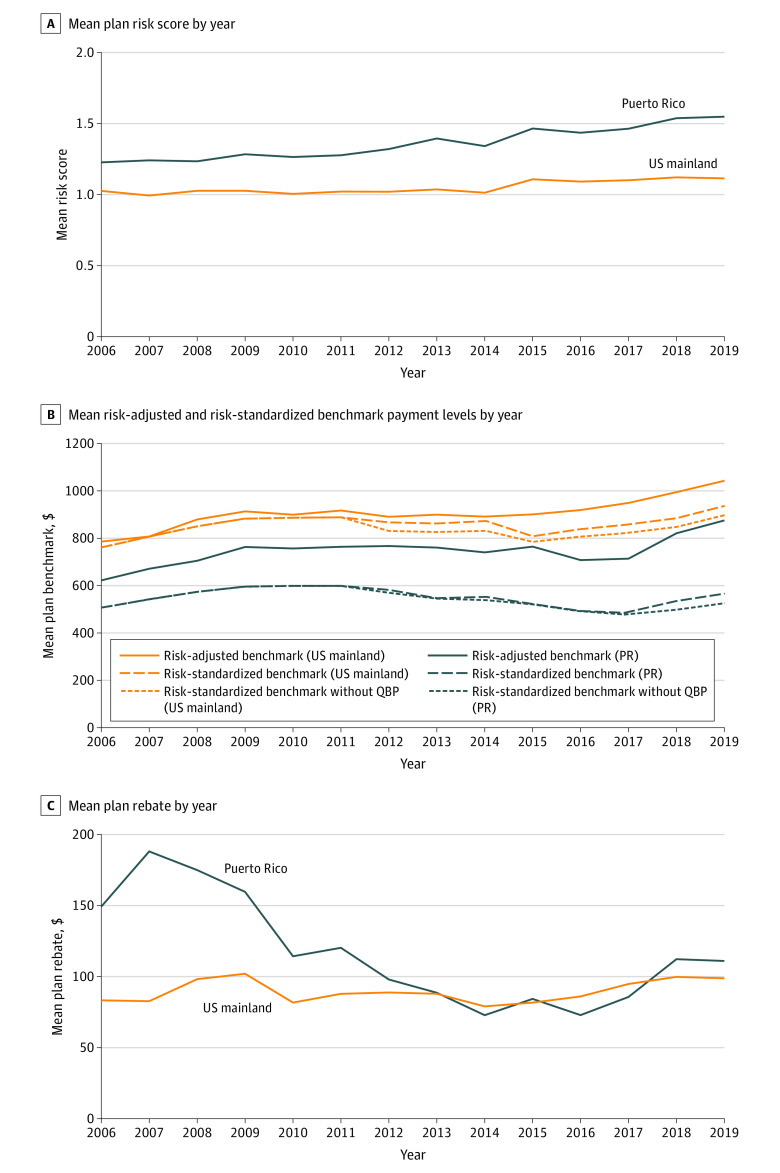
Medicare Advantage Plan Risk Scores, Benchmark Payment Levels, and Rebates in Puerto Rico (PR) and the US Mainland From 2006 to 2019 A, Risk scores were the hierarchical category (HCC) risk scores published in the Centers for Medicare & Medicaid Services (CMS) plan payment data for each plan. Means were not weighted by plan enrollment. B, A risk-standardized benchmark without quality bonus payments (QBPs) was the benchmark that a plan would have had if it had a star rating below the threshold to receive a QBP. Risk-standardized benchmarks were based on the numbers published in CMS rate books inclusive of any QBPs received by the plan that year. Risk-adjusted benchmarks were the plan’s benchmark multiplied by the plan’s CMS HCC risk score. Numbers were not adjusted for inflation. C, Mean rebates were based on plan rebates published in CMS plan payment data. Numbers were not adjusted for inflation. US mainland refers to the 50 US states and Washington, DC.

Medicare Advantage plans in Puerto Rico bid 46% lower for an average-risk beneficiary compared with plans in the US mainland before ACA implementation ($380.01 [95% CI, $373.95-$386.07] vs $700.80 [95% CI, $699.31-$702.30] PBPM) and 43% lower after ACA implementation ($418.70 [95% CI, $412.30-$425.10] vs $732.42 [95% CI, $731.42-$733.41] PBPM). Based on the risk-adjusted bids and benchmarks, plans in Puerto Rico received $168.50 (95% CI, $163.57-$173.42) PBPM in rebates before ACA implementation, exceeding the $92.07 (95% CI, $90.77-$93.37) PBPM among plans in the US mainland. After ACA implementation, rebates in Puerto Rico decreased by 40% to $93.39 (95% CI, $89.51-$97.27) PBPM, which was not significantly different from the $90.02 (95% CI, $89.13-$90.91) PBPM in the US mainland (Table 3 and Figure 1C).

Mean (SD) plan risk scores increased differentially in Puerto Rico, up to 1.55 (0.31) in 2019, compared with mean (SD) plan risk scores in the US mainland (1.15 [0.38]) (Figure 1A). Risk-adjusted benchmarks for plans in Puerto Rico increased at similar rates as in the US mainland after ACA implementation (Figure 1B). Actual plan payments (risk-adjusted bids plus rebates) were 20% lower in Puerto Rico than in the US mainland before ACA implementation ($644.73 [95% CI, $629.83-$659.64] vs $806.60 [95% CI, $802.99-$810.22] PBPM) and 20% lower after ACA implementation ($700.61 [95% CI, $684.51-$716.71] vs $880.69 [95% CI, $877.58-$883.81] PBPM) (Table 3).

### Difference-in-Differences

Risk-standardized benchmarks changed by −$69.85 (95% CI, −$82.04 to −$57.66) PBPM for plans in Puerto Rico compared with plans in the US mainland after ACA implementation (Table 3). Of note, risk-standardized benchmarks in Puerto Rico changed differentially before ACA implementation (mean relative change, −$15.31; 95% CI, −$18.70 to −$11.93); thus, we could not attribute the entire differential change to ACA implementation. Risk-standardized bids for MA plans in Puerto Rico did not change differentially compared with those in the US mainland ($−13.60 [95% CI, $−36.23 to $9.03] PBPM). As a result, rebates in Puerto Rico changed by $−63.70 (95% CI, $−82.73 to $−44.68) PBPM after ACA implementation compared with those in the US mainland. However, risk-adjusted benchmarks and actual plan payments did not differentially decrease for plans in Puerto Rico compared with the US mainland. These findings were similar in sensitivity analyses that included 2010 in the post–ACA implementation period, excluded data from both 2010 and 2011, and included a variable to indicate whether a plan was an SNP (eTable 3 in the Supplement). Models comparing plans in Puerto Rico with plans with the lowest 25% of benchmarks in the US mainland were not valid owing to nonparallel trends before ACA implementation.

### Quality Bonus Payments

**Figure 2.**
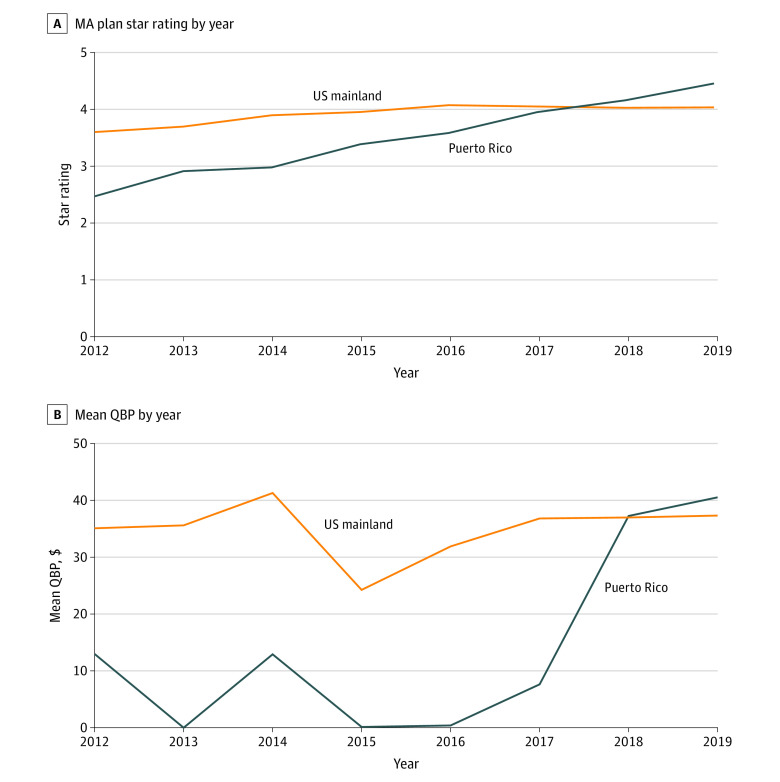
Mean Aggregate Quality Score and Mean Quality Bonus Payment (QBP) for Plans in Puerto Rico and the US Mainland From 2006 to 2019 A, Aggregate quality scores were the Medicare Advantage (MA) star ratings reported by the Centers for Medicare & Medicaid Services (CMS). Plans that were too new or had insufficient data were excluded. Aggregate quality scores were based on data submitted by plans during the 2 previous years. Means were not weighted by plan enrollment. B, Quality bonus payments were estimated using the percentage of plans eligible for QBPs based on MA star ratings and the dollar amount of those QBPs, calculated as the percentage increase in benchmarks associated with QBPs in that year and the risk-standardized benchmarks published in CMS rate books. Numbers were not adjusted for inflation. US mainland refers to the 50 US states and Washington, DC.

Star ratings for plans in Puerto Rico increased from a mean (SD) of 2.47 (0.19) in 2012 to a mean (SD) of 4.46 (0.17) in 2019 (Figure 2A) on a 5-point scale. The mean (SD) ratings in the US mainland improved from 3.58 (0.66) to 4.03 (0.52) during this period. By 2019, 98% of plans in Puerto Rico had a score of 4.0 or higher (the threshold to qualify for the maximum quality bonus payment) compared with 78% of plans in the US mainland. Plans in Puerto Rico received a mean of $41 PBPM from quality bonus payments by 2019, an increase from $0 PBPM in 2016 (Figure 2B). Forty percent of the increase in risk-standardized benchmarks for plans in Puerto Rico from 2017 to 2019 was attributable to increased quality bonus payments to these plans.

## Discussion

This cohort study revealed differences in MA financing in Puerto Rico compared with the US mainland. The study also showed how MA plans in Puerto Rico plausibly responded to ACA implementation. Medicare Advantage plans in Puerto Rico had lower risk-standardized benchmarks than plans in the US mainland, and this gap increased after ACA implementation. The differentially decreased risk-standardized benchmarks in Puerto Rico, which led to differential reductions in rebates for plans in the territory, presumably reduced additional coverage benefits and increased cost sharing for beneficiaries. The reductions in risk-standardized benchmarks were offset by increased risk scores and quality bonus payments in Puerto Rico.

The ACA reduced MA benchmarks for counties in Puerto Rico from 180% to 115% of traditional Medicare FFS spending. Setting benchmarks closer to traditional Medicare spending raised concern about statutory exceptions affecting Puerto Rico and whether FFS spending in Puerto Rico accurately reflected MA beneficiary spending.^24,30^ Several rule changes were made by the CMS after ACA implementation to address these concerns (Table 1), but we were unable to isolate the effects of these policies on final plan payments individually given the data limitations. The persistent disparity in risk-standardized benchmark levels after modifying traditional Medicare payments in Puerto Rico suggests that a large component of the difference in benchmarks was associated with differences in utilization or intensity of care for traditional Medicare beneficiaries in Puerto Rico compared with those in the US mainland.

Our findings suggest that responses by MA plans in Puerto Rico, particularly focusing on diagnosis coding intensity and quality bonus payments, may have contributed more than policy changes by the federal government to counteract the decreased benchmarks. Without large increases in risk scores and quality bonus payments, the gap in plan payments between Puerto Rico and the US mainland would have further widened. Although higher beneficiary risk scores in Puerto Rico may reflect higher rates of chronic medical conditions in the territory and changes by CMS to risk score calculations, the timing of the differential increase in risk scores promptly after ACA implementation and before CMS implemented changes to risk score calculations and the magnitude of the increase suggest that diagnostic coding behavior likely changed.

Although the responses by plans in Puerto Rico partially offset lower federal risk-standardized benchmarks, they may be difficult to sustain. By 2019, 98% of plans in Puerto Rico were receiving the maximum quality bonus payment compared with 78% of plans in the US mainland. The high rate of maximum quality bonus payments in Puerto Rico was associated with a substantial increase in mean star ratings in the territory between 2012 and 2019. This increase was unlikely to have been only associated with adjustments by CMS to star ratings and was unexpected because of the known disparities affecting the Puerto Rican health care system.^4,9,12,40^ The financing gap may widen if quality bonus payments in the US mainland increase compared with those in Puerto Rico. Similarly, mean risk scores for MA plans in Puerto Rico would have to increase to 1.84 to garner similar risk-adjusted benchmarks as MA plans in the US mainland. Despite the decreased risk-standardized benchmarks, several insurers continue to offer MA plans in Puerto Rico, although 5 of the 6 insurers offering plans in Puerto Rico after 2015 only operated in the territory. We did not have data on the underlying costs for these insurers or their plans, but their decision to continue insuring beneficiaries in Puerto Rico suggests that they found the territory to be financially viable.

More research is needed to understand the health care utilization patterns of Medicare beneficiaries in Puerto Rico and whether the funding disparities described in this study affect quality or outcomes for older adults in the territory. To date, there have been few rigorous, claims-based analyses of beneficiary and clinician behavior in Puerto Rico, especially compared with the US mainland.^9,41^ Improved analyses and investments in infrastructure to improve data collection and validation in Puerto Rico may help policy makers develop sustainable, territory-specific policies to address sociodemographic disadvantages and funding disparities in Puerto Rico. Given that the dominant share of Puerto Rico’s health care financing comes from MA, even modest changes to payments in Puerto Rico may be broadly consequential.

### Limitations

This study has several limitations. First, we could not evaluate how health care utilization varied between the US mainland and Puerto Rico at the patient or claims levels. This limitation also prevented us from further exploring the improvement on star ratings in Puerto Rico compared with the US mainland. Second, data limitations prevented adjustment for enrollee demographic characteristics when comparing plans in Puerto Rico with those in the US mainland. Third, we lacked information on the costs of MA plans operating in Puerto Rico, and a detailed analysis of plan benefits was outside the scope of this analysis. Thus, we did not detail how the changes in payments affected plan profitability or generosity. Fourth, we were unable to isolate the effects of individual policies and rule changes to assess how much of the findings were associated with policy changes rather than changes in plan behavior. Of note, the results of our study were robust to sensitivity analyses.

## Conclusions

In this cohort study of MA financing in Puerto Rico compared with the US mainland, risk-standardized benchmarks differentially decreased in Puerto Rico after ACA implementation. This gap in financing was partly offset by increases in risk scores and quality bonus payments for plans in Puerto Rico, some of which may have been associated with recent policy changes. However, federal payments to plans in Puerto Rico still lagged behind those to plans in the US mainland.

## Notes

Supplement. **eFigure 1.** Medicare Advantage Penetration in Puerto Rico and the US Mainland **eFigure 2.** Medicare Advantage Penetration Rates by County in Puerto Rico in 2018 **eFigure 3.** Number of Medicare Advantage Insurers and Plans per County in Puerto Rico and the US Mainland **eFigure 4.** Average Ratio of Medicare Fee-for-Service (FFS) National Payment Amounts to Medicare FFS Payments in Puerto Rico for Selected CPT Codes and GPCI Components for Puerto Rico from 2006 to 2020 **eTable 1.** Study Outcomes, Definitions, and Data Sources **eTable 2.** Comparison of Medicare Fee-for-Service Prices in Puerto Rico and the US Mainland in 2016 **eTable 3.** Results of Sensitivity Analyses for Difference-in-Differences Analysis
