# Supplementary material for: Medicare Advantage Financing and Quality in Puerto Rico vs the 50 US States and Washington, DC
Source: JAMA Health Forum. 2022 Sep 16;3(9):e223073. doi: 10.1001/jamahealthforum.2022.3073 (PMC9482057; doi:10.1001/jamahealthforum.2022.3073)
Supplement: Supplement. — eFigure 1. Medicare Advantage Penetration in Puerto Rico and the US Mainland eFigure 2. Medicare Advantage Penetration Rates by County in Puerto Rico in 2018 eFigure 3. Number of Medicare Advantage Insurers and Plans per County in Puerto Rico and the US Mainland eFigure 4. Average Ratio of Medicare Fee-for-Service (FFS) National Payment Amounts to Medicare FFS Payments in Puerto Rico for Selected CPT Codes and GPCI Components for Puerto Rico from 2006 to 2020 eTable 1. Study Outcomes, Definitions, and Data Sources eTable 2. Comparison of Medicare Fee-for-Service Prices in Puerto Rico and the US Mainland in 2016 eTable 3. Results of Sensitivity Analyses for Difference-in-Differences Analysis [file jamahealthforum-e223073-s001.pdf]

Supplemental Online Content

Roberts T, Song Z. Medicare Advantage financing and quality in Puerto Rico vs the 50 US states and Washington, DC. *JAMA Health Forum*. 2022;3(9):e223073. doi:10.1001/jamahealthforum.2022.3073

eFigure 1. Medicare Advantage Penetration in Puerto Rico and the US Mainland
eFigure 2. Medicare Advantage Penetration Rates by County in Puerto Rico in 2018
eFigure 3. Number of Medicare Advantage Insurers and Plans per County in Puerto Rico and the US Mainland
eFigure 4. Average Ratio of Medicare Fee-for-Service (FFS) National Payment Amounts to Medicare FFS Payments in Puerto Rico for Selected CPT Codes and GPCI Components for Puerto Rico from 2006 to 2020
eTable 1. Study Outcomes, Definitions, and Data Sources
eTable 2. Comparison of Medicare Fee-for-Service Prices in Puerto Rico and the US Mainland in 2016
eTable 3. Results of Sensitivity Analyses for Difference-in-Differences Analysis

This supplemental material has been provided by the authors to give readers additional information about their work.

© 2022 Roberts T et al. *JAMA Health Forum.*

eFigure 1. Medicare Advantage Penetration in Puerto Rico and the US Mainland*

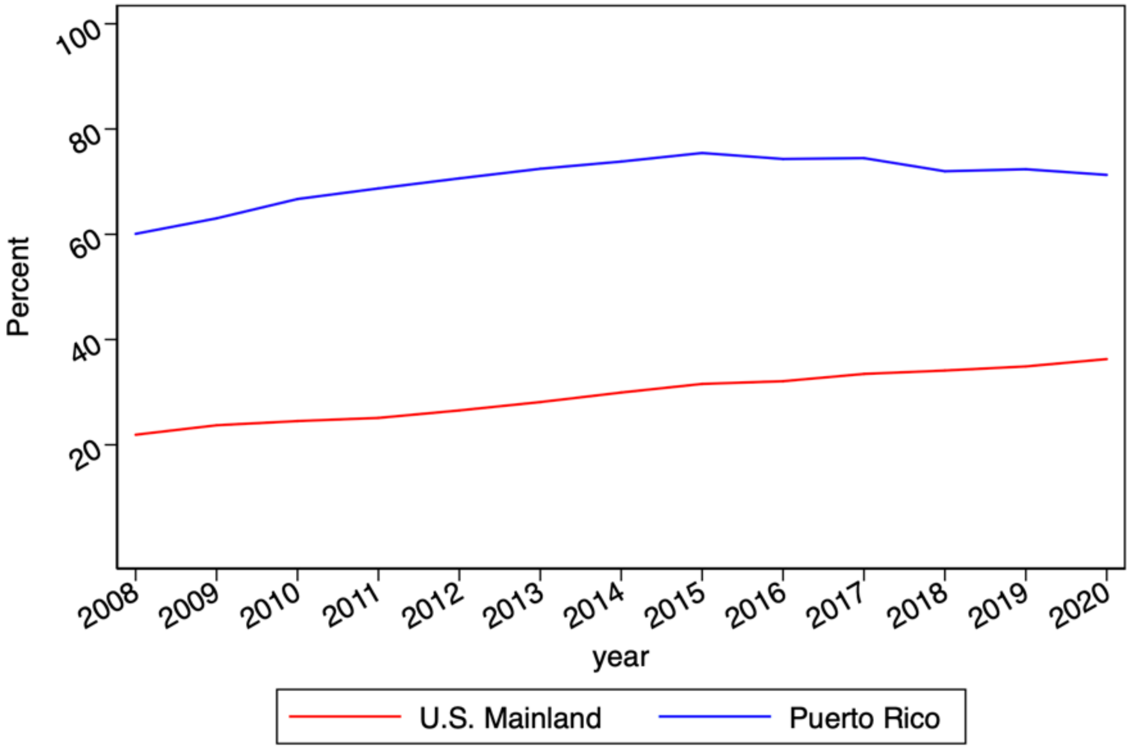

Includes enrollment in all types of Medicare Advantage plans

eFigure 2. Medicare Advantage Penetration Rates by County in Puerto Rico in 2018

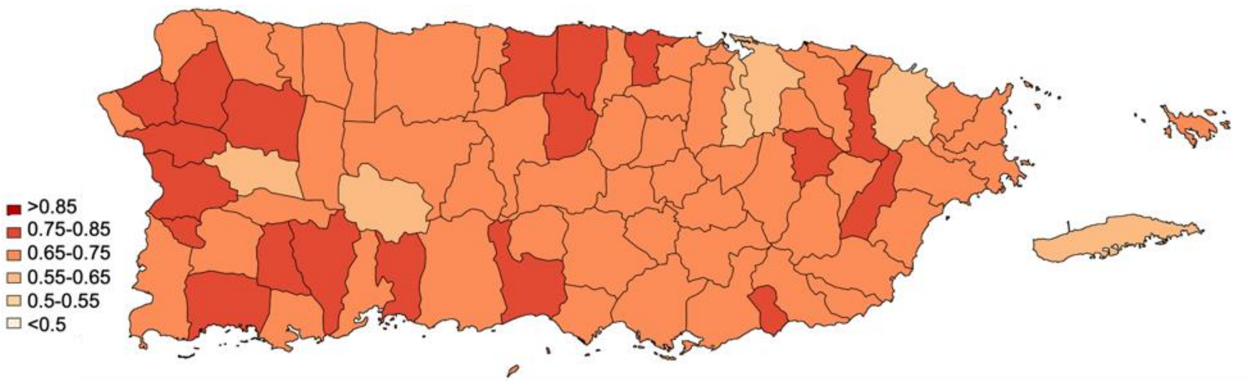

Isla de Desecheo and Isla de Mona are not shown on this map.
Figure based on penetration rates reported by CMS for the year 2018.

© 2022 Roberts T et al. *JAMA Health Forum.*

eFigure 3. Number of Medicare Advantage Insurers and Plans per County in Puerto Rico and the US Mainland

(A) Average Number of Insurers per County in Counties with at least one Medicare Advantage Plan

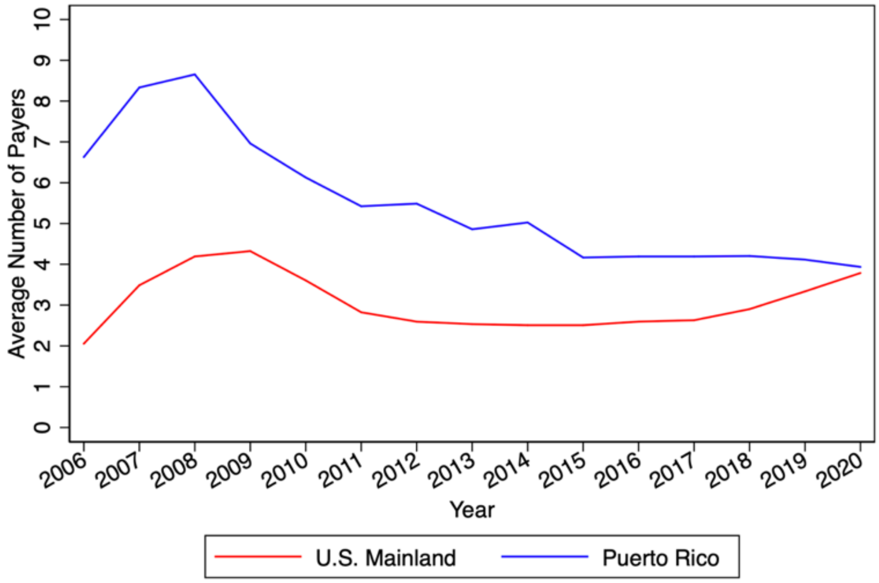

(B) Average Number of Plans per County in Counties with at least One Medicare Advantage Plan

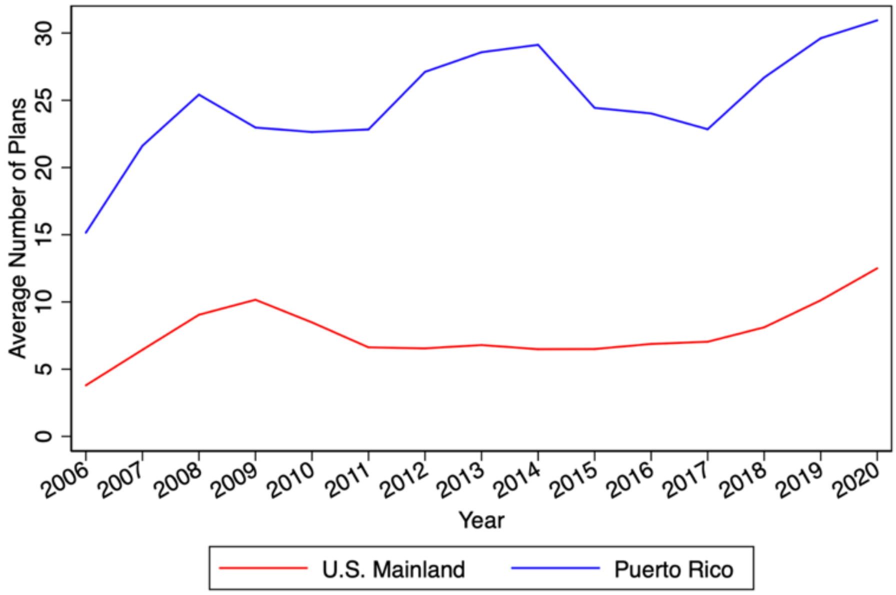

Only includes counties with at least one Medicare Advantage (MA) plan. Nearly all counties in Puerto Rico had at least one MA plan during all years included in the analysis. The percent of counties in the U.S. mainland with at least one MA plan available varied by year.

© 2022 Roberts T et al. *JAMA Health Forum.*

eFigure 4. Average Ratio of Medicare Fee-for-Service (FFS) National Payment Amounts to Medicare FFS Payments in Puerto Rico for Selected CPT Codes and GPCI Components for Puerto Rico from 2006 to 2020

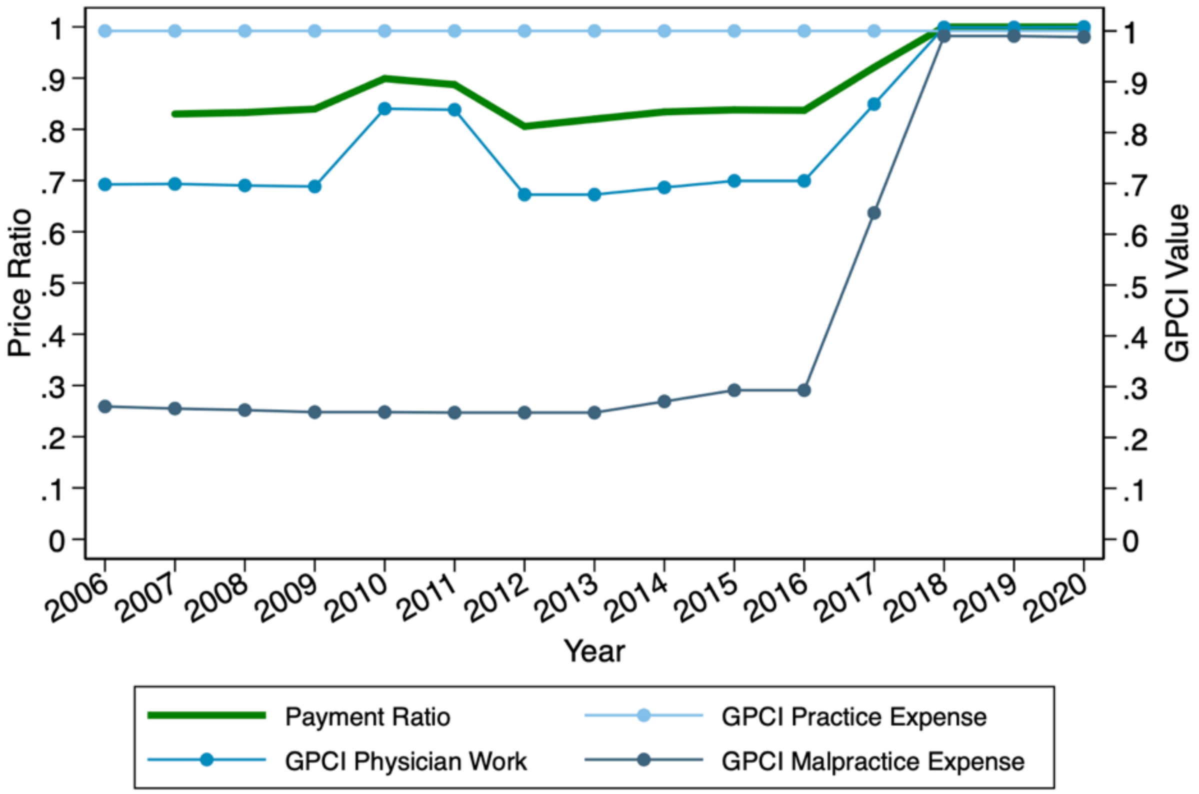

Price ratios were calculated as the ratio of the Medicare Fee-For-Service (FFS) price associated with CPT codes in Puerto Rico to the FFS price in the U.S. mainland based on prices listed in the Medicare fee schedule for each year. To identify the prices, Medicare Administrative Contractor (MAC) 0000000 was used for the U.S. mainland and MAC 0920220 for Puerto Rico. GPCI = Geographic Practice Cost Index. GPCI components were pulled from the Medicare fee schedule for each year using MAC 0000000 for the U.S. mainland and MAC 0920220 for Puerto Rico.

© 2022 Roberts T et al. *JAMA Health Forum.*

eTable 1. Study Outcomes, Definitions, and Data Sources

	Outcome	Definition	Data Sources
**Primary outcomes**	Risk-standardized plan benchmark	The mean benchmark for an average-risk beneficiary enrolled in a plan. This value was calculated as the mean county benchmark across all counties where a plan was active, weighted by the number of enrollees in each county.	CMS rate book (county benchmarks)<br>CMS monthly enrollment reports (county enrollment)
	Risk-standardized bid	The bid submitted by a plan for an average risk beneficiary in each calendar year, as reported in CMS plan payment data	CMS plan payment data
	Plan rebate	The rebate received by a plan in each calendar year, as reported in CMS plan payment data	CMS plan payment data
**Secondary outcomes**	Risk-adjusted benchmark	The mean benchmark for an actual beneficiary enrolled in a plan. This value was calculated as the mean county benchmark across all counties where a plan is active, weighted by the number of enrollees in each county, multiplied by the plan's CMS HCC risk score.	CMS rate book (county benchmarks)<br>CMS monthly enrollment reports (county enrollment)<br>CMS plan payment data (HCC risk scores)
	Risk-adjusted bid	The bid submitted by a plan for an average risk beneficiary in each calendar year multiplied by the plan's CMS HCC risk score	CMS plan payment data
	Actual plan payment	The actual payment received by a plan in each calendar year. This value was calculated as the risk-adjusted bid plus the plan rebate.	CMS plan payment data
	MA star ratings	Overall plan ratings as reports in the annual Medicare star ratings spring releases	Medicare star ratings spring releases

CMS = Center for Medicare and Medicaid Services; HCC = Hierarchical Category Coding; MA = Medicare Advantage

© 2022 Roberts T et al. *JAMA Health Forum.*

eTable 2. Comparison of Medicare Fee-for-Service Prices in Puerto Rico and the US Mainland in 2016

Physician Services (CPT code)	Medicare Fee-for-Service Price, National Payment Amount		Medicare Fee-for-Service Price, Puerto Rico		Ratio
	Non-Facility Price	Facility Price	Non-Facility Price	Facility Price	
**Evaluation and Management**					
New patient office visit (99203)	$108.85	$77.69	$89.52	$67.56	0.83
Emergency department visit (99285)	NA	$175.44	NA	$158.66	0.90
Psychotherapy (90837)	$128.18	$127.11	$120.43	$119.67	0.94
Physical therapy (97110)	$32.58	NA	$27.43	NA	0.84
**Procedures**					
Drainage of abscess (10060)	$118.87	$98.82	$94.77	$80.64	0.81
Hernia repair (49505)	NA	$539.57	NA	$436.74	0.81
Gallbladder removal (47562)	NA	$682.43	NA	$555.12	0.81
Joint injection (20610)	$61.23	$47.26	$49.74	$39.89	0.91
**Imaging and Tests**					
Electrocardiogram (93010)	$8.59	$8.59	$7.71	$7.71	0.90
Chest x-ray (71020)	$27.93	NA	$21.72	NA	0.78
Head computed tomography (70450)	$117.08	NA	$90.63	NA	0.77
Pathology examination (88305)	$74.11	NA	$59.73	NA	0.81
**Mean**					**0.84**

Based on authors' analysis of data from the 2016 Center for Medicare & Medicaid Services (CMS) Physician Fee Schedule.
Current Procedural Terminology (CPT)) codes were used to identify physician services in the CMS Physician Free Schedule. Prices are at the Medicare Administrative Contractor (MAC) level, 0000000 for U.S. mainland and 0920220 for Puerto Rico. Prices are the sum of the non-facility price and the facility price.

© 2022 Roberts T et al. *JAMA Health Forum.*

eTable 3. Results of Sensitivity Analyses for Difference-in-Differences Analysis

Measure	Excluding 2010 & 2011	Including all years	Including special needs plan (SNP) variable
Risk-standardized benchmark[a]	-$72.47 (-86.69 to -58.25)	-$67.88 (-78.58 to -57.18)	-$72.14 (-84.71 to -59.56)
Risk-standardized bid	-$16.92 (-39.28 to 5.43)	-$11.42 (-34.21 to 11.37)	-$9.38 (-31.98 to 13.21)
Rebate	-$68.81 (-87.57 to -50.05)	-$60.29 (-79.88 to -40.71)	$-67.52 (-87.47 to -47.57)
Risk-adjusted benchmark[b]	-$20.11 (-79.69 to 39.46)	-$23.65 (-74.31 to 27.01)	-$4.06 (-40.04 to -31.93)
Risk-adjusted bid[b]	$53.43 (-0.34 to 107.21)	$50.33 (-3.81 to 104.46)	$70.48 (31.02 to 109.95)
Actual plan payment[b]	-$14.54 (-70.27-41.20)	-$9.35 (-60.99-42.30)	$7.18 (-25.75 to 40.11)

[a]*Trends in the risk-standardized benchmark between Puerto Rico and mainland U.S. were not parallel prior to the ACA (average relative change of -$15.31, 95% CI -18.70 to -11.93). Therefore, although the difference increased after the ACA, we are not able to attribute the entire differential change to the ACA.*
[b] *Given these outcomes were risk-adjusted, the model did not further adjust for risk*

© 2022 Roberts T et al. *JAMA Health Forum.*
